# Evaluation of plasma lactate parameters for predicting mortality of septic patients

**DOI:** 10.1016/j.heliyon.2022.e12340

**Published:** 2022-12-10

**Authors:** Mei Wang, Yan Wang, Liu Taotao, Qinyu Zhao, Yangong Chao

**Affiliations:** a Department of Intensive Care Unit, The First Hospital of Tsinghua University, Beijing 100016, China; b Department of Respiratory and Critical Care Medicine, Beijing Jishuitan Hospital, Beijing 100035, China; c Department of Surgical Intensive Care Unit, Beijing Hospital, National Center of Gerontology, Institute of Geriatric Medicine, Chinese Academy of Medical Sciences, Beijing 100730, China; d College of Engineering and Computer Science, Australian National University, Canberra 2600, Australia

**Keywords:** Lactate, Lactate clearance, Sepsis, Mortality, MIMIC-IV

## Abstract

**Objective:**

To compare the accuracy of serum lactate parameters, including lactate peak concentration (LACpeak), lactate time area (LACarea), and lactate clearance (LC) for predicting mortality of the septic patients, and to compare with the predictive accuracy of National Early Warning Score (NEWS) and Sequential Organ Failure Assessment (SOFA) scores.

**Methods:**

This study retrospectively screened the septic patients admitted to the ICU in the Medical Information Mart for Intensive Care IV (MIMIC-IV) from 2008 to 2019. The baseline data and outcomes of patients were gathered. The subjects were divided into the non-survival group and the survival group. SOFA, NEWS, LACpeak, and LACarea were recorded. The LC was calculated 6 h after LACpeak. The above parameters were compared by the T-test and Mann-Whitney U test, and odds ratios were calculated adjusting for age and sex. The receiver operating characteristic curves (ROCs) of subjects were plotted according to SOFA, NEWS, LACpeak, and LACarea within 24h, and LC at 6h of ICU admission. The Areas under the ROC curve (AUCs), sensitivity, and specificity were compared with R version 4.1.1.

**Results:**

1,169 septic patients were involved, and 366 (31.3%) patients died within 28 days. Compared to the survival group, the LACpeak of the non-survival group was higher [4.85 (3.2, 7.9) vs. 3.4 (2.6, 5.25) mmol/L, adjusted odds ratio 1.18, P < 0.001], and the LACarea of the non-survivals was higher than the survivals too [18.44 (10.36, 27.63) vs. 13.65 (9.01, 21.73), adjusted odds ratio 1.03, P < 0.001)]. The LC of the survivals at 6 h after LACpeak was significantly higher than that of the non-survivals [0.26 (0.14.0.42) vs. 0.19 (0.10, 0.33), adjusted odds ratio 0.06, P < 0.01]. Within 24h of ICU admission, the AUCs of mortality prediction in descending order were NEWS [0.73 (0.70, 0.76)], SOFA [0.69 (0.66, 0.73)], LACpeak [0.64 (0.61, 0.68)], and LACarea [0.60 (0.56, 0.63)]. There were 204 patients with 6-hour LC after LACpeak the AUCs of LACarea, LACpeak and LC were 0.73(0.65, 0.80), 0.71(0.62,0.78) and 0.65 (0.56, 0.73), respectively.

**Conclusions:**

The predictive accuracy of LC was not superior to LACpeak and LACarea for the mortality of the septic patients and the predictive value of all the above lactate parameters for mortality maybe not better than SOFA and NEWS.

## 1 Introduction

Sepsis is a life-threatening medical problem, impacting millions of people worldwide and killing between one in three and one in six of those it affects each year [1]. Lactate is known as a relatively simple and inexpensive biomarker to examine at the bedside [2, 3]. It is often used to estimate the severity of septic patients.

Multiple studies [4, 5, 6, 7, 8, 9, 10, 11] suggested that several serum lactate-related parameters, including lactate initial concentration, lactate peak concentration (LACpeak), lactate time area (LACarea), and lactate clearance (LC), were related to the prognosis of the septic patients. Hyperlactatemia is common in several critical illnesses including sepsis and may demonstrate an imbalance between oxygen supply and oxygen consumption [12]. Several studies have confirmed that persistent hyperlactatemia is related to an increased risk of ICU admission and higher mortality [4, 13, 14]. A series of studies have emphasized the prognostic value of LC, but LC cannot suggest the severity of hyperlactatemia [9, 15, 16]. The lactate area is defined as the sum of the area under the curve of serum lactate levels, as it is an index demonstrating the duration and severity of hyperlactatemia [9, 15, 16].

However, the specificity and sensitivity of different lactate parameters for mortality prediction for sepsis were still unclear. The study attempted to assess the prognostic value of lactate parameters including LACpeak, LACarea, and LC, and to compare the predictive accuracy for mortality in sepsis with Sequential Organ Failure Assessment (SOFA) and National Early Warning Score (NEWS).

## 2 Methods

### 2.1 Patient and public involvement

The septic patients admitted to the ICU recorded in the MIMIC-IV database since 2008 to 2019 were identified. The MIMIC-IV database, which includes high-quality medical records of ICU patients, was updated based on the MIMIC-III database. Patients from more than 90 ICUs were included in the MIMIC database [17]. Beth Israel Deaconess Medical Center (Boston, MA) and the Massachusetts Institute of Technology (Cambridge, MA) approved the establishment of this database and obtained consent to the clinical information collection. Therefore, this study was exempted from the informed consent and the ethical approval statement. All patients' data referred to in our study was anonymous.

### 2.2 Inclusion criteria

Patients diagnosed with sepsis within 24 h of ICU admission (met the diagnostic criteria sepsis-3); Age≥18 years old; At least one specimen of arterial lactate was collected when patients were admitted to ICU; Plasma lactate peak concentration (LACpeak) within 24 h, and LACpeak≥2.0 mmol/L; A declined lactate concentration within 24 h next to the LACpeak (LACnext) were recorded, and LACnext < LACpeak.

### 2.3 Exclusion criteria

LACnext ≥ LACpeak; Patients' medical records were insufficient.

## 3 Methods

### 3.1 Grouping

Subjects were divided into the non-survival group and the survival group based on whether they survived within 28 days after the ICU admission.

**Figure 1:**
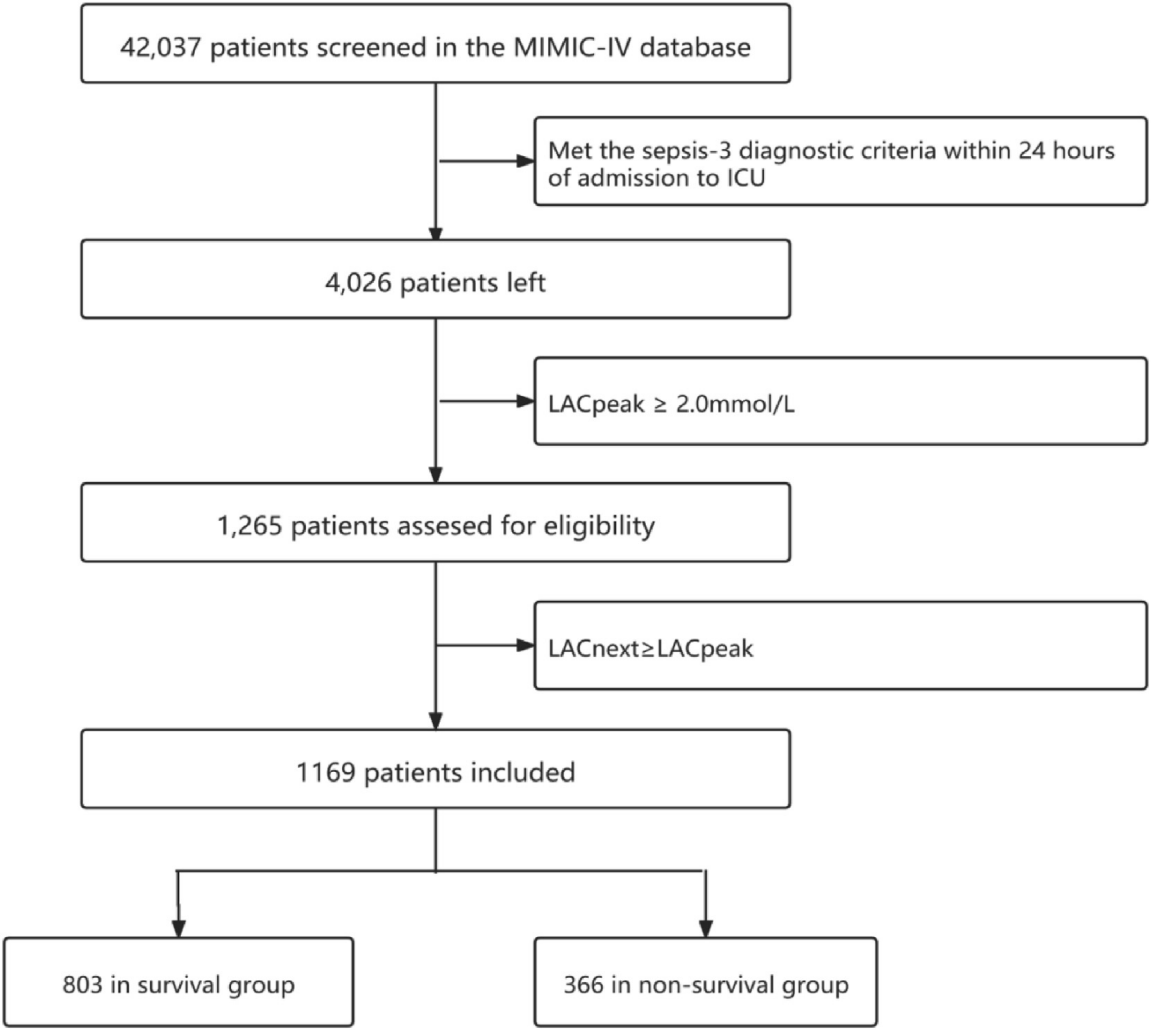
Research flow chart.

The baseline information were gathered, including age, gender, Charlson comorbidity score (CCS), length of stay before ICU admission, vital signs and laboratory test results, etc. The NEWS and SOFA score were recorded. The outcomes were recorded, including 28-day mortality, mechanical ventilation, as well as the length of stay in the ICU and in the hospital. The research flow chart was shown in Figure 1.

The LACpeak within 24 h of ICU admission, the LACnext followed by LACpeak, and the time gap between LACpeak and LACnext were recorded. The LC was calculated 6 ± 1 h after the LACpeak. The calculation formulas of LC and LACarea were as follows:

lactate clearance (LC)=(LACpeak - LACnext)/LACpeak×100%

2 lactate time area (LACarea)=(LACpeak + LAC next)×time gap/

The accuracy of plasma lactate parameters, including LACpeak, LACarea, and LC, and scoring systems of NEWS and SOFA in predicting 28-day mortality were evaluated. The receiver operating characteristic curves (ROCs) were plotted, as well as the areas under the ROC Curves (AUCs), were calculated. The odds ratios of LACpeak, LACarea, the SOFA, and NEWS scores independently associated with 28d mortality were calculated by adjusting for age and sex.

### 3.2 Statistical analysis

The included patients' characteristics were described by descriptive statistics. Variables with normal distributions were shown as means (SD), where independent samples t-test was used to compare. Non-normally distributed variables were presented as medians [IQR] and were compared by the Mann-Whitney U test. Categorical variables were reported as percentages and the Chi-squared test or Fisher's exact test was used. After drawing the ROC curve, the cut-off value was determined according to the Youden index, and the positive predictive value, negative predictive value, specificity, and sensitivity were calculated. SPSS 20.0 and R version 4.1.1 were used to perform the statistical analyses. Figures were produced by Prism 8.0, and p ≤ 0.05 was considered statistically significant.

## 4 Results

### 4.1 Baseline data

Of the 42,037 inpatient records screened, 4,026 inpatients met the diagnostic criteria of sepsis-3, 1,265 had a LACpeak higher than 2.0 mmol/L within 24h, and 1,169 had a LACnext lower than LACpeak in the following 24h. 803 (68.7%) patients survived and 366 (31.3%) patients died within 28 days of ICU admission.

**Figure 2:**
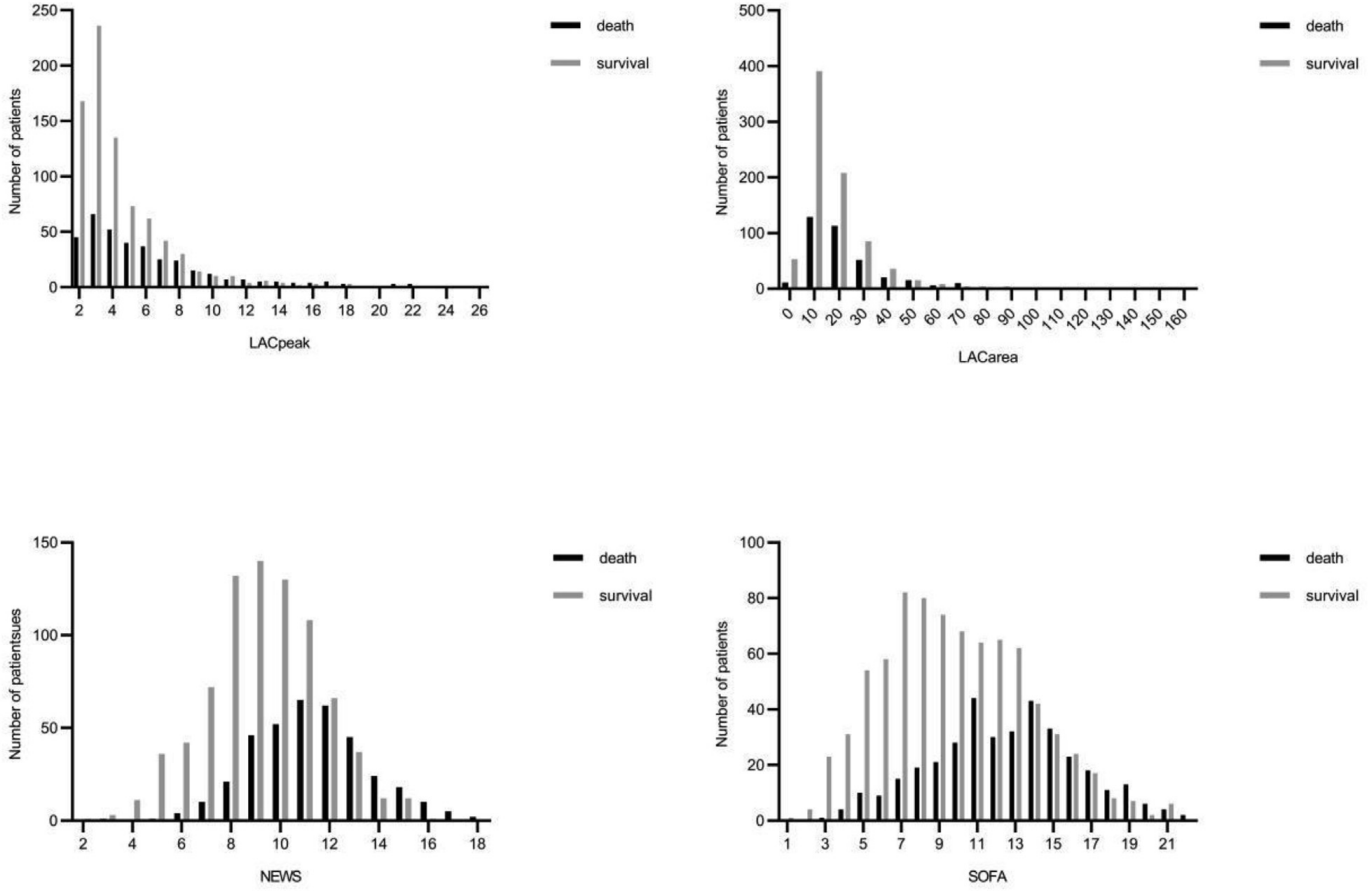
Distribution of LACpeak, LACarea, NEWS, and SOFA in the survival group and the non-survival group.

No statistically significant differences were shown in age, gender, body mass index (BMI), CCS, accounts of white blood cells (WBC), mechanical ventilation, use of positive vasoactive agents between the survival group and in the non-survival group (all P > 0.05). The proportion of sepsis shock in the non-survival group was significantly higher than in the survival group (80.05% vs. 51.68%, P < 0.001). Meanwhile, there were statistically significant differences in SOFA (adjusted odds ratio 1.22, P < 0.001) and NEWS (adjusted odds ratio 1.48, P < 0.001). (Shown in Table 1 and Figure 2.).

**Table 1:** The baseline data of septic patients.

	Total n = 1169	The survival group n = 803	The non-survival group n = 366	*P*-value
Age, mean (SD)	64.56 (16.38)	64.86 (16.45)	63.91 (16.25)	0.358
Male, n (%)	495 (42.34%)	332 (41.34%)	163 (44.54%)	0.337
BMI, mean (SD)	29.4 (8.35)	29.5 (8.2)	29.3 (8.6)	0.786
Sepsis shock, n (%)	708 (%)	415 (51.68%)	293 (80.05%)	<0.001
Charleson comorbidity score, median [Q1, Q3]	6.0 (4.0, 8.0)	6.0 (4.0, 8.0)	6.0 (4.0, 8.0)	0.501
Baseline data				
GSC score, mean (SD)	8.67 (4.21)	9.91 (3.8)	5.87 (3.8)	<0.001
Temperature, mean (SD) (°C)	37.59 (1.06)	37.68 (0.93)	37.40 (1.29)	<0.001
Systolic pressure, mean (SD) (mmHg)	146.83 (25.94)	149.94 (23.80)	144.42 (30.01)	<0.001
Mean arterial pressure (MBP), mean (SD) (mmHg)	107.67 (25.94)	106.83 (223.69)	109.51 (30.09)	<0.001
Heart rates, mean (SD) (bpm)	114.49 (22.75)	113.12 (22.39)	117.50 (23.26)	0.003
Respiratory rates, mean (SD) (bpm)	30.54 (7.17)	30.16 (7.10)	31.37 (6.82)	<0.001
PH, mean (SD)	7.39 (0.09)	7.41 (0.08)	7.37 (0.10)	<0.001
White blood cells, mean (SD) (∗10^9^/L)	19.52 (16.71)	19.63 (18.24)	19.27 (12.76)	0.675
Creatinine, mean (SD) (mg/dl)	2.27 (1.77)	2.13 (1.72)	2.59 (1.83)	<0.001
Length of stay before being admitted to ICU, median [Q1, Q3](d)	1.95 (0.92,14.11)	2.17 (0.97, 15.35)	1.7 (0.79, 6.78)	0.034
APS-III, median [Q1, Q3]	75 (56, 98)	67 (50, 87)	93 (74, 116)	<0.001
SOFA, mean (SD)	11 (8, 13)	9 (7, 12)	13 (10,15)	<0.001
NEWS, mean (SD)	10 (8, 12)	9 (8, 11)	11 (10, 13)	<0.001
Therapeutic measures				
Mechanical ventilation, n (%)	325 (27.80%)	219 (27.27%)	106 (28.96%)	0.550
Positive vasoactive agents, (n, %)	158 (13.52%)	100 (12.45%)	58 (15.85%)	0.116
Length of stay in the hospital, median [Q1, Q3] (d)	11 (6, 20)	14 (8, 23)	5 (2,12)	<0.001
Length of stay in ICU, median [Q1, Q3] (d)	4.25 (2.25, 8.96)	4.67 (2.71, 9.08)	3.65 (1.64, 7.87)	<0.001

### 4.2 Lactate parameters

**Figure 3:**
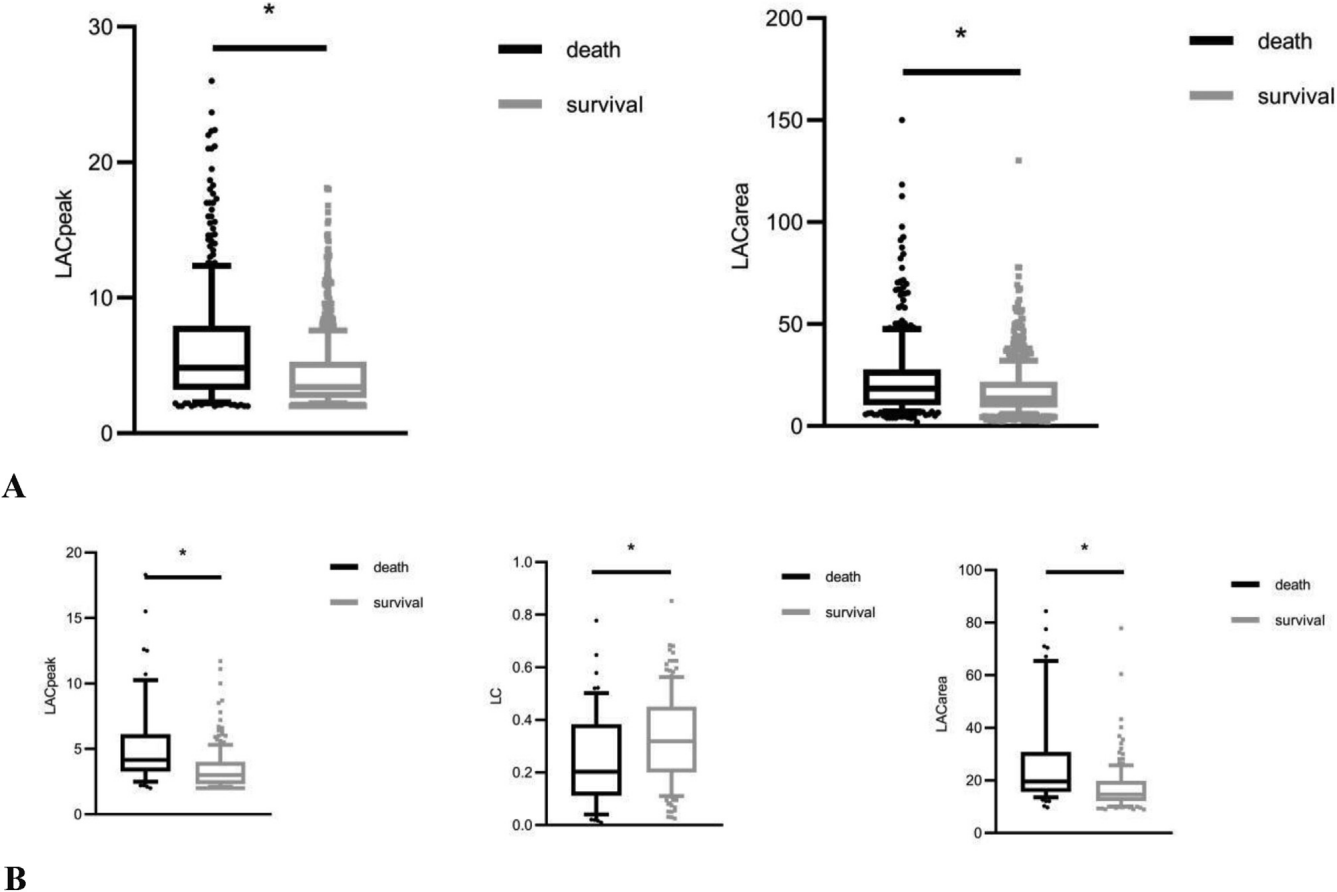
The comparison of the lactate parameters between the two groups. A: The comparison of the two groups according to LACpeak and LACarea within 24h. B: The comparison of the two groups according to LACpeak, LC, and LACarea at 6±1h.

**Figure 4:**
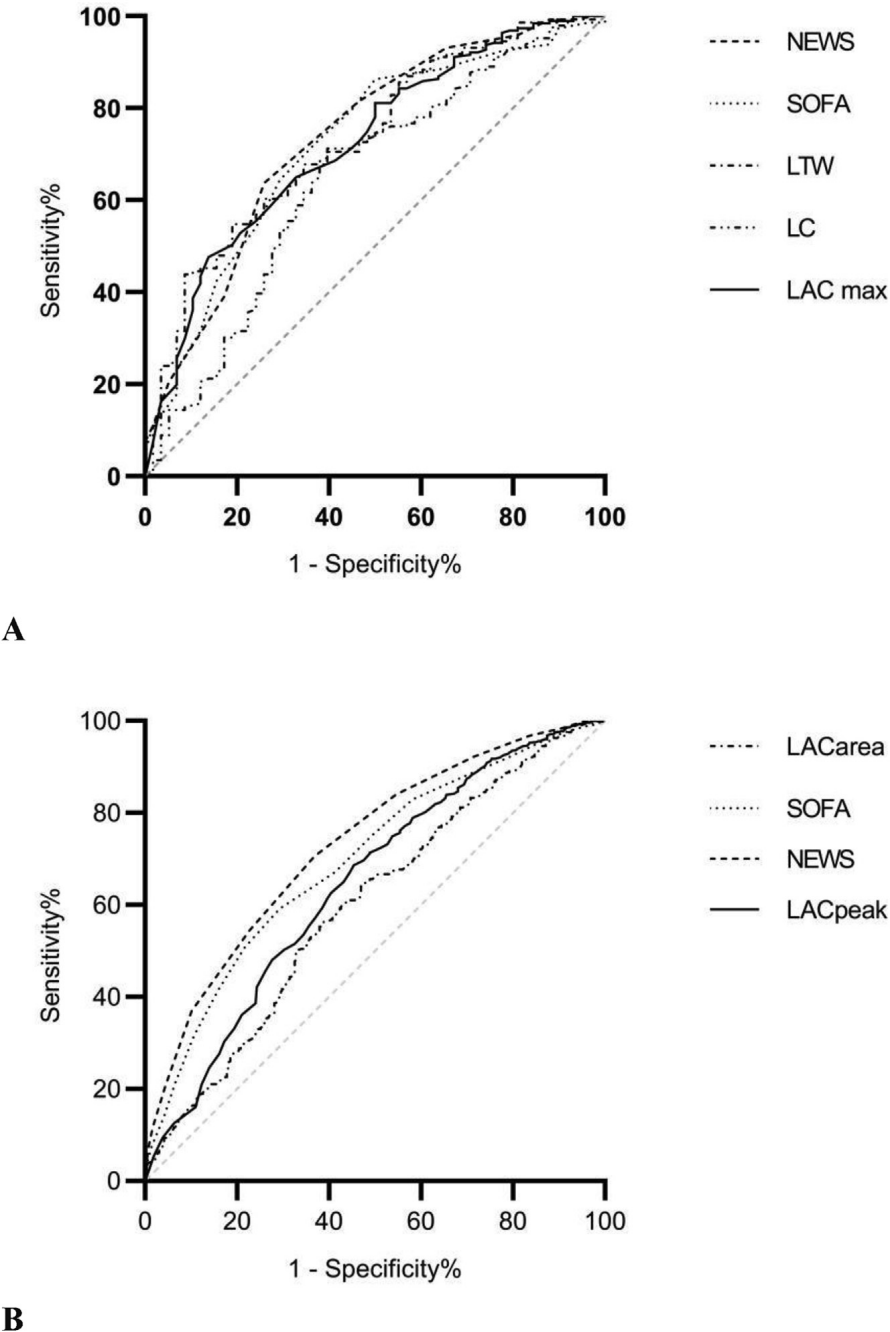
The ROC curves of mortality prediction. A: The ROCs of LACarea, LC, LACpeak, SOFA, and NEWS at 6±1h for 28-day mortality prediction. B: The ROCs of LACarea, LACpeak, SOFA, and NEWS within 24h for 28-day mortality prediction.

Compared to the survival group, the LACpeak within 24h of ICU admission in the non-survival group was higher [4.85 (3.2, 7.9) vs. 3.4 (2.6, 5.25) mmol/L, adjusted odds ratio 1.18, P < 0.001)]. The LACarea of the non-survivals was higher than that of the survivals, too [18.44 (10.36, 27.63)vs. 13.65 (9.01, 21.73), adjusted odds ratio 1.03, P < 0.001)]. The LC 6 h after the LACpeak of the survivals was higher than that of the non-survivals [0.26 (0.14.0.42) vs. 0.19 (0.10, 0.33), adjusted odds ratio 0.06, P < 0.01]. (Shown in Tables 2 and 3, Figure 3A, B and Figure 4A, B).

**Table 2:** The comparison of plasma lactate parameters between the two groups.

	Total	The survival group	The non-survival group	*P-*value
1169 patients within 24h of ICU admission				
Number of patients, n	1169	803	366	-
LACpeak, median [Q1, Q3] (mmol/L)	3.8 [2.7, 6.0]	3.4 [2.6, 5.25]	4.85 [3.2, 7.9]	<0.001
LACnext within 24h, median [Q1, Q3] (mmol/L)	2.6 [1.8, 4.5]	2.4 [1.7, 3.7]	3.6 [2.1, 6.6]	<0.001
LAC time gap, median [Q1, Q3] (h)	4.12 [2.55, 6.40]	4.17 [2.6,6.55]	4.07 [2.39, 6.23]	0.138
LACarea, median [Q1, Q3]	15.03 [9.40, 23.43]	13.65 [9.01, 21.73]	18.44 [10.36, 27.63]	<0.001
patients with 6-hour lactate records after LACpeak				
Number of patients, n	204	146	58	-
LACarea at 6±1h, median [Q1, Q3]	16.08 [12.62, 21.60]	14.65 [12.16, 19.90]	19.66 [15.63, 30.89]	0.001
LC at 6±1h, median [Q1, Q3]	0.29 [0.17, 0.44]	0.32 [0.20, 0.45]	0.20 [0.11, 0.38]	<0.001

**Table 3:** Logistic regression models showing variables independently associated with 28-day mortality adjusting for age and sex.

	Odds Ratio	95% CI	*P-*value
1169 patients within 24h of ICU admission			
SOFA	1.22	1.17, 1.26	<0.001
NEWS	1.48	1.39, 1.58	<0.001
LACpeak	1.18	1.14, 1.23	<0.001
LACarea	1.03	1.02, 1.03	<0.001
204 patients with 6-hour lactate records after LACpeak			
SOFA	1.23	1.13, 1.34	<0.001
NEWS	1.58	1.32, 1.89	<0.001
LACpeak	1.40	1.20, 1.64	<0.001
LACarea	1.07	1.04, 1.10	<0.001
LC	0.06	0.01, 0.42	<0.01

### 4.3 The predictive accuracy of the lactate parameters for 28-day mortality in septic patients

Within 24h of ICU admission, the AUC of NEWS for predicting the mortality in septic patients was 0.73 (0.70, 0.76), higher than SOFA [0.69 (0.66, 0.73)], LACpeak [0.64 (0.61, 0.68)], and LACarea [0.60 (0.56, 0.63)], all P < 0.05.

The AUC of 6-hour LC for predicting the mortality in septic patients was 0.65 (0.56, 0.73), which is not significantly lower than that of LACpeak [0.71 (0.62,0.78)] and LACarea [0.73 (0.65, 0.80)], both P > 0.05 (Shown in Table 4 and Figure 3A, B-4A, B.).

**Table 4:** The comparison of the lactate parameters for predictive accuracy of 28d mortality in patients with sepsis.

	AUC (95% CI)	*P-*value	Cutoff value	Youden index	Sensitivity	Specificity	positive likelihood ratio	negative likelihood ratio	Positive predictive value	Negative predictive value
1,169 patients within 24h										
NEWS	0.73 (0.70, 0.76)	Compared with the other 3 parameters	11	0.34	63 (58, 68)	71 (68, 74)	2.147506	0.5223784	50 (45, 54)	81 (78, 84)
SOFA	0.69 (0.66, 0.73)	<0.05	11	0.30	71 (66, 75)	59 (56, 63)	1.732449	0.4942249	44 (40, 48)	82 (79, 85)
LACpeak	0.64 (0.61,0.68)	<0.001	4.6	0.23	55 (49, 59)	69 (65, 71)	1.741261	0.660984	44 (40, 49)	77 (74, 80)
LACarea	0.60 (0.56,0.63)	<0.001	15.3	0.17	61 (56, 66)	56 (53, 60)	1.400153	0.6892621	39 (35, 43)	76 (72, 79)
204 patients 6±1h after the LACpeak										
NEWS	0.74 (0.66,0.81)	0.112	10	38	74 (62, 85)	63 (56, 71)	2.04229	0.4060067	45 (34, 54)	86 (80, 92)
SOFA	0.72 (0.64,0.80)	0.149	14	36	50 (37, 63)	86 (80, 91)	3.65	0.5793651	59 (44, 73)	81 (75, 87)
LACarea	0.73 (0.65, 0.80)	0.132	15.1	36	81 (71, 91)	55 (47, 63)	1.765826	0.350502	42 (32, 51)	88 (81, 95)
LACpeak	0.70 (0.63,0.78)	0.358	3	32	86 (77, 95)	46 (38, 54)	1.593191	0.3005661	39 (30, 47)	90 (83, 96)
LC	0.65 (0.56, 0.73)	Compared with the other 4 parameters	0.23	32	60 (47, 72)	71 (63, 78)	2.037767	0.5809019	46 (34, 57)	82 (75, 88)

## 5 Discussion

The study retrospectively screened more than 40,000 medical records in the MIMIC-IV database, and the 28-day mortality of the 1,169 septic patients enrolled was 31.3%, which is consistent with previous literature reports [1, 5, 8, 11].

Lactate clearance is one of the commonly used resuscitation indexes in the treatment of sepsis, because of its simplicity of calculation and availability. Several studies support the view that an LC rate≤10% within 6 h of resuscitation can decrease the mortality of septic patients [3, 18, 19]. However, our study found that the AUC of LC 6 h after the LACpeak for mortality prediction was less than 0.7. There was no statistical difference in the AUC of LC compared with LACpeak and LACarea, but it may be related to the small sample size of patients included in this subgroup. The prognostic accuracy of LC was not better than LACpeak and LACarea when it was used as the single predictive factor for mortality. It was because the LC rate can only reflect the declined proportion of lactate, but cannot reflect the LACpeak and the subsequently declined value. For example, in the same time interval, compared with the LACpeak decreased from 5 mmol/L to 4.5 mmol/L, the LACpeak decreased from 10 mmol/L to 9 mmol/L in patients with sepsis is obviously a more critical condition, even though they have the same LC rate. Moreover, the LC of 10% is the target value of 6 h of resuscitation, but the target value of LC in other time intervals is unclear, which is not conducive to dynamic monitoring of the disease condition.

The study did not analyze the lactate initial concentration which was widely used in previous studies [15, 20, 21]. It is because the course of illness and the occasion of the individuals' admission to the ICU is different, and the collection time of the initial lactate concentration is also different. Moreover, the initial lactate concentration cannot reflect the treatment efficacy. Thus, we didn't choose patients with the initial lactate concentration, but patients with LACpeak within 24h of admission to the ICU instead as the research population, to analyze the prognostic value of lactate concentration. The LACpeak is identified as an endpoint of resuscitation, an index of disease severity, and a predictor of prognosis [3, 5]. In our study, the LACpeak within 24h of ICU admission of the non-survivals was much higher than that of the survivals [4.85 (3.2, 7.9) mmol/L vs. 3.4 (2.6, 5.25) mmol/L, P < 0.001]. In the ICU setting, the AUCs of LACpeak for mortality prediction of seriously ill patients varied from 0.53 [19] to 0.86 [22]. In this study, the AUC of LACpeak for mortality prediction was more than 0.7, which indicated moderate prognostic performance.

The LACarea consisted of two lactate absolute values at different times and the time gap between them. Persistent elevation of lactate is related to poor prognosis, and conversely, a declined lactate (or LC) during resuscitation is identified as an independent predictor for decreased mortality [4, 23].

The LACarea can not only reflect the severity of sepsis but also indicate the effect of resuscitation treatment. Therefore, this parameter may be more valuable for the prediction of mortality. The data in this study supported the above hypothesis, and it was found that LACarea was superior to LACpeak and LC when used to judge the severity of the disease. The AUC of the LACarea was more than 0.7 when only compared with the LC 6 h after the LACpeak. However, due to the complex calculation method of LACarea, its clinical application and research reports are not as extensive as the LACpeak and LC.

In our study, more than 1000 patients were included with a LACpeak within 24 h after ICU admission, and more than 200 patients had a declined lactate concentration 6 h after the LACpeak. For the above two groups of different subjects, the maximum values of NEWS and SOFA scores within 24 h were used for evaluation, and their AUC areas were slightly higher than 0.7. When lactate parameters 6 h after the LACpeak were analyzed, the AUC of LACarea for mortality prediction increased from 0.6 to more than 0.7, indicating a significant difference between the two groups. This may be related to the lower heterogeneity of patients with lactate parameters 6 h after LACpeak and the better prognostic accuracy of data.

In clinical conditions, many scoring tools predict sepsis outcomes based on baseline data, regardless of resuscitation effect. The SOFA score is a systematic and comprehensive evaluation of the patients' organ function, which is mainly applicable to patients with sepsis and is not as complicated as the Acute Physiology and Chronic Health Evaluation (APACHE) score [24], while NEWS is not dependent on laboratory examination and is mainly calculated based on vital signs, which is widely used in the emergency department [25, 26]. Therefore, we choose to compare with the above two scores in this study. We found that the prognostic value of LACarea was not superior to SOFA and NEWS. It is because although LACarea and LC reflect the resuscitation effect, the severity of the disease cannot be fully evaluated by a single parameter. Therefore, the combination of LACpeak or LACarea with the NEWS score may be more valuable for mortality prediction in septic patients.

Limitation: Limited to the retrospective study, the septic patients included in this study have a long period, during which the sepsis treatment plans would be quite different. It would lead to different resuscitation treatment outcomes. Meanwhile, patients with LAC >2.0 mmol/L but with elevated LAC after resuscitation were excluded, which can affect the LACpeak and the LC rates and may skew the results. In addition, as there were only about 200 patients with 6-hour LC rates recorded, the small sample size resulted in a P-value >0.05 when comparing the AUC of LC with other parameters, showing no statistical difference. Larger sample size studies are needed to clarify the statistical differences in the future.

## 6 Conclusion

The predictive accuracy of LC was not superior to LACpeak and LACarea for the mortality of septic patients and the predictive value of all the above lactate parameters for mortality maybe not better than SOFA and NEWS.

## Declarations

### Author contribution statement

Mei Wang conceived and designed the experiments, performed the experiments, analysed and interpreted the data, and wrote the paper. Yan Wang helped to analyse and interpreted the data, and wrote the paper. Yangong Chao helped to conceive and designed the experiments, interpreted the data, and revise the paper.

### Funding statement

This research did not receive any specific grant from funding agencies in the public, commercial, or not-for-profit sectors.

### Data availability statement

Data included in article/supp. material/referenced in article.

### Declaration of interest's statement

The authors declare no competing interests.

### Additional information

No additional information is available for this paper.
